# Derivation and validation of a screening tool for stroke-associated sepsis

**DOI:** 10.1186/s42466-023-00258-4

**Published:** 2023-07-13

**Authors:** Sebastian Stösser, Lisa Kleusch, Alina Schenk, Matthias Schmid, Gabor C. Petzold

**Affiliations:** 1 Division of Vascular Neurology, Department of Neurology, University Hospital Bonn, Venusberg-Campus 1, 53127 Bonn, Germany; 2 Institute of Medical Biometry, Informatics and Epidemiology, Medical Faculty, University of Bonn, Bonn, Germany; 3 German Center for Neurodegenerative Diseases (DZNE), Bonn, Germany

**Keywords:** sepsis, Ischemic stroke, Patient outcome assessment, Infections, Organ dysfunction scores

## Abstract

**Background:**

Post-stroke infections may cause sepsis, which is associated with poor clinical outcome. Sepsis is defined by life-threatening organ dysfunction that can be identified using the Sequential Organ Failure Assessment (SOFA) score. The applicability of the SOFA score for patients not treated on an intensive care unit (ICU) is limited. The aim of this study was to develop and validate an easier-to-use modification of the SOFA score for stroke patients.

**Methods:**

Using a registry-based cohort of 212 patients with large vessel occlusion stroke and infection, potential predictors of a poor outcome indicating sepsis were assessed by logistic regression. The derived score was validated on a separate cohort of 391 patients with ischemic stroke and infection admitted to our hospital over a period of 1.5 years.

**Results:**

The derived Stroke-SOFA (S-SOFA) score included the following predictors: National Institutes of Health stroke scale ≥ 14, peripheral oxygen saturation < 90%, mean arterial pressure < 70 mmHg, thrombocyte count < 150 10^9^/l and creatinine ≥ 1.2 mg/dl. The area under the receiver operating curve for the prediction of a poor outcome indicating sepsis was 0.713 [95% confidence interval: 0.665–0.762] for the S-SOFA score, which was comparable to the standard SOFA score (0.750 [0.703–0.798]), but the prespecified criteria for non-inferiority were not met (p = 0.115). However, the S-SOFA score was non-inferior compared to the SOFA score in non-ICU patients (p = 0.013).

**Conclusions:**

The derived S-SOFA score may be useful to identify non-ICU patients with stroke-associated sepsis who have a high risk of a poor outcome.

**Supplementary Information:**

The online version contains supplementary material available at 10.1186/s42466-023-00258-4.

## Introduction

Infections such as pneumonia and urinary tract infections are associated with an unfavorable clinical outcome after stroke [1, 2]. Pneumonia occurs in 8 to 12% of stroke patients with dysphagia being the most important risk factor [3]. Urinary tract infections occur in 8 to 19% of stroke patients and are usually catheter-associated [4]. In stroke patients treated on an ICU, the spectrum of infections also includes surgical site infections, meningitis and ventriculitis if a craniotomy was performed or devices, such as external ventricular drains or intracranial pressure monitors, were placed [5].

Stroke-associated infections may lead to sepsis which is characterized by a dysregulated host response to the infection and subsequent organ dysfunction leading to high mortality and morbidity [6]. Previous studies reported sepsis in 2 to 13% after stroke [7–12]. These incidence numbers vary based on the studied cohorts and applied methodology: The lowest incidence was reported in a study that used data from a healthcare policy registry and included all patients with a diagnosis of ischemic stroke, while the highest incidence was reported in a study that specifically collected data on sepsis in a more severely affected cohort of patients with large vessel occlusion stroke [7, 12]. Regarding the association of sepsis with clinical outcome, the results of previous studies are rather consistent. The odds for a poor outcome, indicated by in-hospital mortality in most studies, were increased around twofold in patients with sepsis compared to stroke patients without sepsis [7–12].

According to the Sepsis-3 definition, the life-threatening organ dysfunction that defines sepsis can be identified using the Sequential Organ Failure Assessment (SOFA) score (acute increase of ≥ 2 points) [6]. The SOFA score assesses the function of six organ systems (central nervous system, respiration, cardiovascular, coagulation, liver, renal) using a score of 0 to 4 for each organ [13]. The SOFA score was developed for patients treated on an ICU and thus requires several parameters, such as arterial blood gases, that are usually not available in patients treated outside an ICU. Most stroke patients, however, are treated on a stroke unit or normal ward and not on an ICU. This limits the use of the SOFA score in such cases. The easier-to-use quick SOFA (qSOFA) score was proposed as screening tool for sepsis in patients outside an ICU [14]. However, we observed in a previous study that the qSOFA score had a low specificity in stroke patients, in particular since the items “altered mentation” and “respiratory rate ≥ 22/min” were often found to be positive in stroke patients without sepsis or infection [12]. Thus, there is a need for a clinically practical and specific tool to facilitate the diagnosis of sepsis in stroke patients.

The aim of this study was to develop and validate a modification of the SOFA score that can detect organ dysfunction associated with poor outcome and thus sepsis in stroke patients with infections as validly as the original SOFA score, but is easier to use in practice.

## Methods

### Derivation of the modified SOFA score

A previously described, registry-based cohort of 212 patients with large vessel occlusion stroke and stroke-associated infection was used as training data to derive the modified SOFA score [12]. This cohort included patients treated at the University Hospital Bonn between 2016 and 2020 that were included in the German Stroke Registry-Endovascular Therapy (GSR-ET), an ongoing, prospective multicenter registry of stroke patients undergoing endovascular therapy [15].

The modified SOFA score was derived in three steps based on the procedure that was used to develop the qSOFA score [14]:

Firstly, alternative predictors for each subcategory of the SOFA score that were available in the training data and thus reflect parameters that are routinely available in patients treated on a stroke unit were evaluated. Additionally, predictors of systemic inflammatory reactions were tested. Based on previous work, it was assumed that a poor outcome would be more common in stroke patients with sepsis compared to stroke patients with an infection without septic course and, therefore, that patients with sepsis may be discriminated from patients with an uncomplicated infection by the occurrence of a poor outcome [8, 12]. This assumption was chosen in analogy to the one used to develop the qSOFA score [14]. A poor outcome was defined as a score of 5 or 6 on the modified Rankin scale (mRS) at 3 months after stroke. This definition was chosen over mortality because it better discriminates stroke patients with sepsis from stroke patients with an infection without septic course based on previous work (odds ratio [OR] 3.5 for mRS 5–6 versus 1.8 for mortality) [12]. Thus, the OR for a poor outcome was determined for each predictor by logistic regression. Parameters that predicted a poor outcome significantly were carried on to the next step.

Secondly, metric predictors were dichotomized by defining optimal cut-offs using the Youden index on the area under the receiver operating characteristic curve (AUC) for a poor outcome [16]. The cut-off values were rounded to the nearest whole and to the nearest whole divisible by 5 for blood pressure values.

Thirdly, combinations of these predictors were assessed by multivariable logistic regression in order to determine the goodness of fit of these models using Nagelkerke’s R^2^ [17]. At each of those three steps, considerations regarding clinical practicability were taken into account in addition to the statistical analysis.

### Sample size calculation for the validation cohort

The sample size for the validation cohort was calculated based on the following hypothesis: The AUC for the prediction of a poor outcome of the modified SOFA score is non-inferior to the AUC of the SOFA score on the first day of stroke-associated infection.

Based on clinical experience, the non-inferiority margin was set to an AUC difference (modified SOFA minus SOFA) of -0.06. Using this margin and assuming an AUC difference of 0.06 (obtained from the derivation cohort), a sample size of 354 patients was found sufficient to reject the null hypothesis “${AUC}_{modified SOFA}-{AUC}_{SOFA }< -0.06$” with a power of 80% at the one-sided 2.5% significance level (using the formula of DeLong for the standard error of the AUC difference and assuming a poor outcome in 30% of the patients [18]). Details regarding the sample size calculation are given in supplemental material 1. As around 950 patients with ischemic stroke are treated in our hospital per year and around 30% of those may suffer from stroke-associated infections, we decided to acquire data of over the period of 1.5 years (presumed n = 428).

### Data collection for the validation cohort

For the validation cohort, ischemic stroke patients admitted to our hospital between January 2021 and June 2022 were retrospectively identified by diagnostic code. Patients with a diagnosis of infection documented in the medical record by the treating clinician were included. Exclusion criteria were incomplete medical records, a diagnosis of infection before the onset of stroke and/or admission, lack of consent to the prospective evaluation of outcome at 3 months, and failure to complete the follow up at 3 months. The onset of infection was defined as the earliest day on which a diagnosis of infection was documented in the medical record or antibiotic therapy was started. Clinical and laboratory data were retrospectively collected from medical records.

SOFA score variables and potential alternative predictors were determined at admission, on the first day of infection and on the following day. The worst documented value on the first day of infection was recorded. If the partial arterial oxygen pressure (P_a_O_2_) was not available, the P_a_O_2_/Fraction of inspired oxygen (F_I_O_2_) ratio was substituted by the peripheral oxygen saturation (S_p_O_2_)/F_I_O_2_ ratio as described previously [12, 19]. Other missing variables were assumed to be zero. Sepsis was defined as an increase in total SOFA score of two points or more over the baseline score at admission within the period from the onset of infection to the following day according to the Sepsis-3 definition [6]. Further, pneumonia was evaluated according to the Pneumonia in Stroke Consensus (PISCES) criteria and the Centers for Disease Control and Prevention (CDC) criteria for ventilator-associated pneumonia, respectively [20, 21]. Urinary tract infections were evaluated according to CDC criteria [22]. The stroke etiology was evaluated according to TOAST criteria [23], and comorbidities using the Charlson Comorbidity Index [24]. Treatment limitations that exceeded do-not-resuscitate/do-not-intubate (DNR/DNI) orders were summarized as further treatment limitations. These included a wider spectrum of therapy decisions ranging from comfort measures only with cessation of all curative efforts on one side to a decision to continue, but not escalate the current therapy on the other side. The clinical outcome at 3 months was evaluated using the mRS. mRS values were collected from the medical record if they had been evaluated and documented at a follow-up visit. If not available from the record, the mRS was evaluated prospectively by a telephone interview.

### Assessment of test validity

As primary measure of test validity, it was tested if the AUC for the prediction of a poor outcome indicative of sepsis of the modified SOFA score was non-inferior to the AUC of the SOFA score using a non-inferiority margin of 0.06. Secondary measures of test validity included the AUC for the prediction of in-hospital mortality and mortality within 3 months as well as subgroup analyses (patients without treatment limitations, patients treated on an ICU, patients not treated on an ICU).

### Statistics

Statistical analyses were performed using the Statistical Package for Social Sciences version 27.0.0.0 (IBM SPSS Statistics, Armonk, N.Y., USA) and the R language and environment for statistical computing (version 4.1.0).

Continuous variables were summarized by medians with interquartile ranges (Q1-Q3). Ordinal variables were presented as absolute numbers and frequencies. In the derivation cohort, ORs with 95% confidence intervals (CI) for single predictors were determined by univariable logistic regression and were considered as being statistically significant if 95% CI did not include 1. Multivariable logistic regression models were evaluated using Nagelkerke’s R^2^. In the validation cohort, AUC values with 95% CI were determined using the DeLong method [18, 25].

To test non-inferiority of the modified SOFA score to the SOFA score, we considered the difference in AUC values. 95% CI for the difference ${AUC}_{modified SOFA}-{AUC}_{SOFA}$ were constructed according to the DeLong approach [18, 25]. The null hypothesis of inferiority of the modified SOFA score was rejected if the lower bound of the CI for the difference was larger than − 0.06 or, equivalently, if the respective one-sided p-value was smaller than 0.025. Further information on the non-inferiority test is given in supplemental material 2.

## Results

### Derivation of the modified SOFA score

In order to derive the modified SOFA score, potential alternative predictors for a poor clinical outcome at 3 months after stroke indicating sepsis were evaluated in the training data set. Table 1 shows the ORs for a poor clinical outcome for all analyzed predictors. We proceeded with the following predictors to the next step as they significantly predicted a poor clinical outcome: the National Institutes of Health Stroke Scale (NIHSS) at 24 h, the NIHSS subcategory score for level of consciousness, the Glasgow coma scale, representing central nervous system (CNS) function; the respiratory rate and the S_p_O_2_, representing respiratory function; the thrombocyte count, representing coagulation. Mean arterial pressure, representing cardiovascular function, and creatinine, representing renal function, were also carried on to the next step, even though the association with a poor outcome was not significant, as arterial hypotension and renal failure are hallmarks of sepsis.

**Table 1:** Evaluation of alternative predictors of SOFA score subcategory variables and predictors of systemic inflammation

SOFA score subcategory	Predictor	Odds Ratio [95% confidence interval]	Carried on to the next step
Central nervous system	NIHSS at admission	1.040 [0.992, 1.092]	No
	NIHSS at 24 h	1.133 [1.075, 1.194]	Yes
	NIHSS subcategory “level of consciousness”	1.831 [1.325, 2.530]	Yes
	Glasgow coma scale	1.714 [1.313, 2.239]	Yes
	Initial ASPECTS	1.021 [0.807, 1.291]	No
Respiration	Respiratory rate	1.052 [1.004, 1.102]	Yes
	Peripheral oxygen saturation	0.926 [0.872, 0.983]	Yes
	Oxygen flow rate	1.091 [0.984, 1.210]	No
Cardiovascular	Mean arterial pressure	0.984 [0.967, 1.001]	Yes
	Systolic blood pressure	1.008 [0.990, 1.027]	No
	Heart rate	1.008 [0.994, 1.022]	No
	Lactate	1.125 [0.708, 1.789]	No
Coagulation	Thrombocyte count	0.992 [0.988, 0.997]	Yes
Liver	Bilirubin	1.067 [0.376, 3.028]	No
Renal	Creatinine	1.033 [0.758, 1.407]	Yes
	Urea	1.011 [0.994, 1.028]	No
	Urine output in 24 h	1.000 [0.999, 1.001]	No
Systemic inflammation	Temperature	1.313 [0.808, 2.132]	No
	Leukocyte count	1.028 [0.962, 1.099]	No
	c-reactive protein	1.004 [0.998, 1.010]	No
	procalcitonin	0.940 [0.799, 1.107]	No

The optimal cut-offs for the remaining predictors determined using the Youden index are shown in Supplemental Table 1. For the mean arterial pressure, the established cut-off (< 70 mmHg) from the SOFA score was included additionally. For the thrombocyte count and creatinine, the determined cut-offs were still included in the normal range of these values, so we decided to use the established cut-offs from the SOFA score (thrombocytes < 150 10^9^/l, creatinine ≥ 1.2 mg/dl) instead.

Different combinations of the derived predictors were tested for their goodness of fit. The results are shown in Supplemental Table 2. The models 1, 3, 7 and 9, which all included the NIHSS at 24 h, the mean arterial pressure (MAP), the thrombocyte count and creatinine, performed equally well indicated by a Nagelkerke’s R^2^ of 0.315, 0.312, 0.310, and 0.310, respectively. Model 9 was chosen as final model since it included the MAP with the established cut-off of < 70 mmHg and the S_p_O_2_ over the respiratory rate as measure for respiration. Thus, the modified SOFA score included the following dichotomized parameters: NIHSS ≥ 14, S_p_O_2_ <90%, MAP < 70 mmHg, thrombocyte count < 150 10^9^/l and creatinine ≥ 1.2 mg/dl. We termed this score Stroke-SOFA (S-SOFA) score. Its application in comparison to the SOFA score is illustrated in Table 2. The AUC of the S-SOFA score for the prediction of a poor outcome at 3 months indicating sepsis was 0.754 compared to 0.714 for the SOFA score in the derivation cohort.

**Table 2:** Comparison of S-SOFA and SOFA score parameters

Organ system	S-SOFA score cutoff indicating a score of 1	SOFA score cutoffs indicating a score of 1; 2; 3; 4, respectively
CNS	NIHSS ≥ 14	GCS 13–14; 10–12; 6–9; <6
Respiration	S_p_O_2_ <90%	P_a_O_2_/F_I_O_2_, mmHg < 400; <300; <200; <100
Cardiovascular	MAP < 70 mmHg	MAP < 70 mmHg; vasopressor dosage, µg/kg/min dopamine ≤ 5 or dobutamine (any dose); dopamine 5–15, epinephrine/norepinephrine ≤ 0.1; dopamine > 15, epinephrine/norepinephrine > 0.1
Coagulation	Thrombocyte count < 150 10^9^/l	Thrombocyte count, 10^9^/l < 150; <100; <50; <20
Liver	-	Bilirubin, mg/dl 1.2–1.9; 2.0-5.9; 6.0-11.9; >12.0
Renal	Creatinine ≥ 1.2 mg/dl	Creatinine, mg/dl 1.2–1.9; 2.0-3.4; 3.5–4.9 or urine output < 500 ml/d; >5.0 or urine output < 200 ml/d
Score range	0–5	0–24
Relevant increase	≥ 2 over baseline	≥ 2 over baseline

Abbreviations: CNS = central nervous system, MAP = mean arterial pressure, SOFA = Sequential Organ Failure Assessement, S-SOFA = Stroke-SOFA

### Characterization of the validation cohort

For the validation cohort, 1403 cases with ischemic stroke consecutively admitted to our hospital over 1.5 years were screened for a stroke-associated infection. 850 cases were excluded because there was no evidence of infection, and 162 cases were excluded for other exclusion criteria. Thus, 391 patients with stroke-associated infection were included in the validation cohort. The details of the inclusion process are shown in Supplemental Fig. 1. Baseline data, stroke characteristics and treatment, stroke-associated infection, and clinical outcome of the validation cohort are shown in Table 3.

**Table 3:** Characterization of the validation cohort (391 patients with ischemic stroke and stroke-associated infection)

Baseline data	
Age, year, median (Q1-Q3)	80 (71–86)
Sex, female	220 (56.3)
Arterial hypertension	335 (85.7)
Dyslipidemia	265 (67.8)
Atrial fibrillation	200 (51.2)
Smoking	78 (19.9)
Diabetes mellitus	125 (32.0)
Charlson comorbidity index score, median (Q1-Q3)	2 (1–3)
Premorbid mRS, median (Q1-Q3)	1 (0–3)
**Stroke characteristics and treatment**	
NIHSS score at admission, median (Q1-Q3)	10 (5–17)
ASPECTS at admission, median (Q1-Q3)	7 (5–9)
Occlusion of large intracranial arteries	203 (51.9)
Middle cerebral artery, M1 segment	88 (43.3)
Middle cerebral artery, M2 segment	52 (25.6)
Intracranial internal carotid artery	35 (17.2)
Basilar artery	13 (6.4)
Other	15 (7.4)
Stroke etiology	
Cardioembolism	155 (39.6)
Large artery arteriosclerosis	61 (15.6)
Small vessel disease	19 (4.9)
Other determined etiology	17 (4.3)
Undetermined etiology	139 (35.5)
Intravenous thrombolysis	97 (24.8)
Endovascular therapy	145 (37.1)
General anesthesia for endovascular therapy	137 (95.1)
Symptomatic intracranial hemorrhage after intravenous thrombolysis or endovascular therapy	18 (9.5)
Treatment limitations	
DNR	147 (37.6)
DNI	132 (33.8)
Time from admission to DNR/DNI order, days, median (Q1-Q3)	2 (1–5)
Further treatment limitations including comfort measures only	79 (20.2)
Time from admission to further treatment limitations, days, median (Q1-Q3)	7 (3–15)
**Stroke-associated infection**	
Time from admission to diagnosis of infection, days, median (Q1-Q3)	3 (2–6)
Source of infection	
Pneumonia (clinical diagnosis)	186 (47.6)
Pneumonia (according to PISCES criteria)	135 (34.5)
Urinary tract infection (clinical diagnosis)	170 (43.5)
Urinary tract infection (according to CDC criteria)	18 (4.6)
Other	31 (7.9)
Undetermined	41 (10.5)
COVID-19	17 (4.3)
Evidence of a pathogenic organism in body fluid cultures	198 (50.6)
Antibiotic therapy	370 (94.6)
Time from diagnosis of infection to antibiotic therapy, hours, median (Q1-Q3)	1.4 (0.13–4.12)
Sepsis (diagnosis according to Sepsis-3 definition)	129 (33.0)
**Clinical outcome at discharge**	
NIHSS, median (Q1-Q3)	5 (3–11)
mRS, median (Q1-Q3)	4 (3–5)
Barthel index, median (Q1-Q3)	25 (10-62.5)
Length of stay, d, median (Q1-Q3)	13 (7–19)
ICU treatment	95 (24.3)
Death	72 (18.4)
**Clinical outcome at 3 months**	
mRS, median (Q1-Q3)	5 (3–6)
mRS 0	4 (1.0)
mRS 1	21 (5.4)
mRS 2	20 (5.1)
mRS 3	56 (14.4)
mRS 4	87 (22.3)
mRS 5	60 (15.3)
Death (mRS 6)	142 (36.3)
Functional independency (mRS 0–2)	45 (11.5)
Unfavourable outcome (mRS 4–6)	289 (74.1)
Poor outcome (mRS 5–6)	202 (51.7)

Results are presented as n (%) unless indicated otherwise for certain variables. Abbreviations: ASPECTS = Alberta Stroke Programme Early Computed Tomography Score, CDC = Centers for Disease Control and Prevention, COVID-19 = coronavirus disease 2019, DNI = Do not intubate, DNR = Do not resuscitate, ICU = intensive care unit. mRS = modified Rankin Scale, NIHSS = National Institutes of Health Stroke Scale, PISCES = Pneumonia in Stroke Consensus

Other occlusions of large intracranial arteries include vertebral artery, P1/2 segment of the posterior cerebral artery, and A1 segment of the anterior cerebral artery

Notably, the patients of the validation cohort had a median age of 80 (Q1-Q3: 71–86) years, a high frequency of atrial fibrillation (51.2%) and large vessel occlusion stroke (51.9%), and consequently a high frequency of endovascular therapy (37.1%). Treatment limitation orders were frequent (Do-not-resuscitate 37.6%; Do-not-intubate 33.8%; further treatment limitations including comfort measures only: 20.2%).

**Fig. 1:**
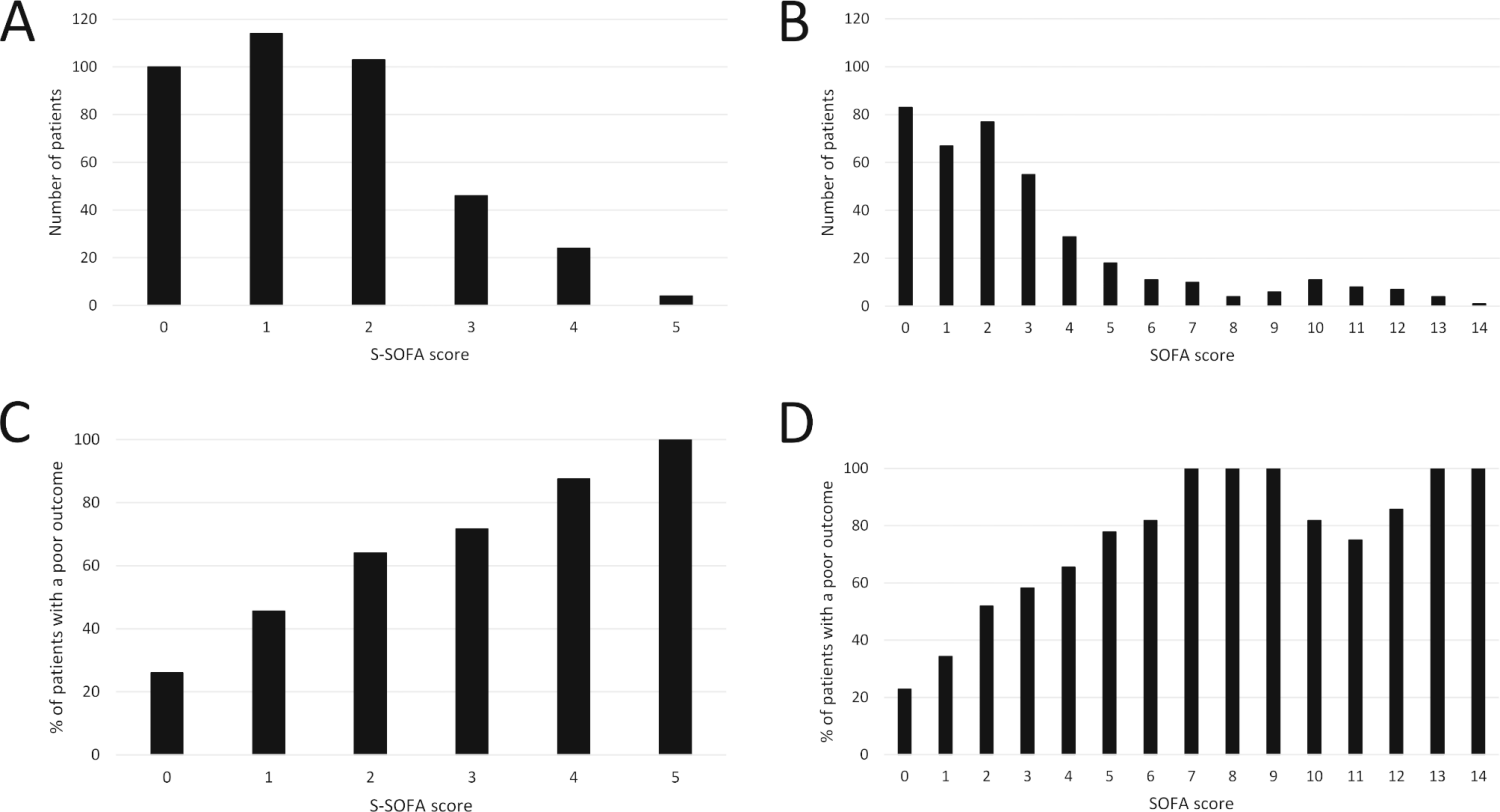
Distribution of Sequential Organ Failure Assessment (SOFA) scores and Stroke-SOFA (S-SOFA) scores in the validation cohort at the onset of infection (**A**: S-SOFA score, **B**: SOFA score). Frequencies of patients with a poor outcome (bedridden or dead) three months after stroke for S-SOFA (**C**) and SOFA scores (**D**) in the validation cohort

Stroke-associated infection was diagnosed at a median of 3 (Q1-Q3: 2–6) days after admission. The major sources of infection were pneumonia (47.6%) and urinary tract infections (43.5%) based on clinical diagnoses. The frequencies were lower when standardized diagnostic criteria were applied (pneumonia: 34.5%; urinary tract infection: 4.6%). Sepsis according to the Sepsis-3 definition was present in 33.0%. The distribution of SOFA and S-SOFA scores at the onset of infection is shown in Fig. 1A-B.

The clinical outcome at 90 days was poor (mRS 5–6) in 51.7%. The median mRS was 5 (Q1-Q3: 3–6). 36.3% of patients had died within 90 days. Only 11.5% demonstrated functional independency (mRS 0–2) at 90 days.

Compared to patients without treatment limitations, patients with treatment limitations were significantly older (median age of 84 vs. 76 years), displayed a higher burden of disability prior to the stroke (median premorbid mRS 2 vs. 0), had a higher frequency of atrial fibrillation (62.0% vs. 43.4%), had more severe strokes (median NIHSS at admission 16 vs. 7), a higher frequency of pneumonia (62.6% vs. 36.8%) and sepsis (46.6% vs. 23.2%), a shorter time from admission to the onset of infection (median of 2 vs. 3 days) and a worse functional outcome at all points in time (e.g. mortality at 3 months 71.8% vs. 11.0%). Detailed data of patients with and without treatment limitations are shown in Supplemental Table 3.

### Test validity of the modified SOFA score

The percentages of patients with a poor outcome for each SOFA and S-SOFA score point value are shown in Fig. 1C-D. An increase of the S-SOFA score of 2 points or more over baseline was observed in 96 (24.6%) patients as compared to 129 (33.0%) for the SOFA score. The OR [95% CI] for a poor outcome of these S-SOFA score positive patients was 2.572 [1.578, 4.129] as compared to 4.532 [2.833, 7.250] for SOFA score positive patients.

AUCs of both scores and their differences for the whole cohort and subcohorts are shown in Table 4 and Supplemental Table 4. In the whole cohort, the AUC for the prediction of a poor outcome at 3 months indicating sepsis was 0.713 [95% CI: 0.665, 0.762] for the S-SOFA score compared to 0.750 [0.703, 0.798] for the SOFA score in the validation cohort at diagnosis of infection. The lower limit of the 95% CI for the difference of the AUCs was − 0.075, marginally lower and thus missing the prespecified non-inferiority margin of -0.060. The S-SOFA score was also not found to be non-inferior to the SOFA score regarding prediction of death during hospitalization, death within 3 months, and an unfavourable outcome (defined by an mRS 4–6) in the whole cohort. If patients with treatment limitations were excluded from the analysis, non-inferiority of the S-SOFA score compared to the SOFA score could not be shown for the analyzed outcome parameters. In the subgroup of patients treated on an ICU, non-inferiority of the S-SOFA score could not be shown either, regardless if patients with treatment limitations were excluded or not.

**Table 4:** Area under the receiver operating curve (AUC) of S-SOFA and SOFA scores for the prediction of different outcome measures and their difference with 95% confidence intervals in the validation cohort and several subgroups (no treatment limitations, ICU treatment, non-ICU treatment)

Predicted outcome	S-SOFA score	SOFA score	Difference	p-value for non-inferiority
Full (n = 391)				
Poor outcome at 3 months	0.713 [0.665, 0.762]	0.750 [0.703, 0.798]	-0.037 [-0.075, 0]	0.115
Death during hospitalization	0.727 [0.666, 0.788]	0.789 [0.728, 0.849]	-0.062 [-0.12, -0.004]	0.526
Death within 3 months	0.684 [0.632, 0.736]	0.724 [0.672, 0.775]	-0.040 [-0.081, 0.002]	0.169
No treatment limitations (n = 228)				
Poor outcome at 3 months	0.688 [0.615; 0.761]	0.707 [0.633; 0.781]	-0.019 [-0.073; 0.035]	0.068
Death during hospitalization	0.863 [0.688; 1.038]	0.988 [0.965; 1.01]	-0.125 [-0.279; 0.029]	0.795
Death within 3 months	0.670 [0.567; 0.772]	0.653 [0.534; 0.772]	0.017 [-0.064; 0.098]	0.032
ICU treatment (n = 95)				
Poor outcome at 3 months	0.770 [0.681, 0.860]	0.780 [0.668, 0.893]	-0.010 [-0.131, 0.111]	0.209
Death during hospitalization	0.678 [0.571, 0.785]	0.756 [0.659, 0.854]	-0.078 [-0.192, 0.035]	0.625
Death within 3 months	0.679 [0.575, 0.782]	0.758 [0.661, 0.856]	-0.080 [-0.195, 0.035]	0.633
ICU treatment and no treatment limitations (n = 50)				
Poor outcome at 3 months	0.770 [0.648; 0.893]	0.732 [0.59; 0.874]	0.038 [-0.111; 0.187]	0.098
Death during hospitalization	0.831 [0.593; 1.07]	0.940 [0.829; 1.051]	-0.109 [-0.247; 0.029]	0.756
Death within 3 months	0.743 [0.527; 0.958]	0.854 [0.697; 1.011]	-0.111 [-0.29; 0.067]	0.713
Non-ICU treatment (n = 296)				
Poor outcome at 3 months	0.686 [0.628, 0.745]	0.703 [0.645, 0.761]	-0.017 [-0.055, 0.021]	0.013
Death during hospitalization	0.739 [0.655, 0.822]	0.699 [0.601, 0.798]	0.039 [-0.024, 0.103]	0.001
Death within 3 months	0.678 [0.616, 0.739]	0.693 [0.631, 0.756]	-0.015 [-0.056, 0.025]	0.016
Non-ICU treatment and no treatment limitations (n = 178)				
Poor outcome at 3 months	0.636 [0.544; 0.729]	0.644 [0.55; 0.737]	-0.008 [-0.07; 0.054]	0.049
Death during hospitalization	-	-	-	-
Death within 3 months	0.647 [0.527; 0.767]	0.597 [0.46; 0.733]	0.051 [-0.033; 0.134]	0.005

If the lower limit of the 95% confidence interval of the difference was greater than or equal to -0.060 (or, equivalently, if the p-value was less than 0.025), the null-hypothesis of inferiority was rejected, and non-inferiority of the S-SOFA score was assumed

Abbreviations: ICU = intensive care unit, SOFA = Sequential Organ Failure Assessment, S-SOFA = Stroke-SOFA

For patients not treated on an ICU, however, the S-SOFA score was found to be non-inferior to the SOFA score regarding the prediction of poor outcome at 3 months (AUC_S−SOFA_: 0.686 [0.628, 0.745] versus AUC_SOFA_: 0.703 [0.645, 0.761]; lower 95% CI for the difference − 0.017), death during hospitalization (0.739 [0.655, 0.822] versus 0.699 [0.601, 0.798]; 0.039), and death within 3 months (0.678 [0.616, 0.739] versus 0.693 [0.631, 0.756]; -0.015). The same held true for death within 3 months when patients not treated on an ICU and without treatment limitations were analyzed. The AUC for death during hospitalization could not be determined for this subgroup as there were no events.

## Discussion

In this study, we developed and validated a modification of the SOFA score, termed S-SOFA, for stroke patients. We found that the S-SOFA score predicted a poor outcome and thus possible sepsis in stroke patients with infections not treated on an ICU as validly as the SOFA score in our cohort. The main advantage of using the S-SOFA score over the SOFA score is that it is easier to apply, in particular in non-ICU patients. While the SOFA score assesses the function of six organ systems using a score of 0 to 4 for each organ system, the S-SOFA score assesses five organ systems using a score of 0 or 1 for each organ system [13]. An increase of the SOFA score of 2 points or more over baseline is used to diagnose sepsis according to the Sepsis-3 definition [6]. The S-SOFA score is used the same way: An increase of 2 points or more predicts a poor outcome after stroke-associated infection indicating sepsis.

In the S-SOFA score, CNS function is evaluated by the NIHSS score as this predicted a poor outcome better than the Glasgow coma scale used in the SOFA score. NIHSS scores are routinely assessed in patients treated on a stroke unit, and hence these data are readily available. The NIHSS at 24 h was a better predictor than the NIHSS at admission. This was not surprising since the NIHSS may improve due to intravenous thrombolysis or endovascular therapy after admission, so the NIHSS at 24 h better reflects actual stroke severity. In the S-SOFA score, the NIHSS was evaluated at the onset of infection and not necessarily after 24 h to capture any changes in the neurological status that occurred due to septic encephalopathy.

Respiratory function is assessed by S_p_O_2_ in the S-SOFA score compared to the P_a_O_2_/F_I_O_2_ quotient in the SOFA score. The P_a_O_2_/F_I_O_2_ quotient can be assessed reliably in ventilated patients with an arterial access only. The majority of stroke patients are neither ventilated nor have an arterial access, so the respiratory subcategory of the SOFA score cannot be assessed in most stroke patients. The S_p_O_2_ used in the S-SOFA score, however, can easily be measured by pulse oximetry. Another respiratory parameter evaluated was the respiratory rate. It predicted a poor outcome equally well as S_p_O_2_. However, changes in respiration not due to infections are frequent in stroke patients, which makes the respiratory rate an unspecific predictor [12, 26]. Thus, S_p_O_2_ was chosen to assess respiratory function in the S-SOFA score.

While the evaluated measures of cardiovascular function did not predict a poor outcome independently, the analysis of different models showed that including the MAP improved the models over not including any measure of cardiovascular function. The cut-off of 70 mmHg is the same as in the SOFA score. Thrombocyte count and creatinine to assess coagulation and renal function, respectively, use the same cut-off as the SOFA score as well. These can be easily assessed by routine laboratory tests. Liver function is assessed in the SOFA score, but not in the S-SOFA score as it was not an independent predictor of a poor outcome and disturbance of liver function occurs very infrequently in stroke patients [12].

The S-SOFA score was derived on a cohort of patients with stroke-associated infection after large vessel occlusion stroke undergoing endovascular therapy. As this constitutes only a minority of stroke patients encountered in clinical practice, the S-SOFA score was validated in a separate cohort of stroke patients. This cohort included consecutive patients with stroke-associated infection over 1.5 years and, thus, is representative of a general population of stroke patients. The major sources of infection were pneumonia and urinary tract infections as expected.

In our study, the S-SOFA score predicted a poor outcome indicating sepsis as validly as the SOFA score in stroke patients not treated on an ICU. For the whole cohort and other subgroups, we were not able to establish non-inferiority of the S-SOFA score compared to the SOFA score using the prespecified non-inferiority margin of -0.060. However, the mean differences of AUC were rather small, ranging from − 0.125 to 0.051, indicating a similar performance of both scores.

The observation that the S-SOFA score performed best in non-ICU patients in relation to the SOFA score was to be expected. The S-SOFA score only contains items that are available in patients treated on a stroke unit, while some items of the SOFA score are not available in non-ICU patients. In ICU patients, the difference of AUCs was higher than in other subgroups, indicating a possible advantage of the more granular SOFA score in these patients. However, this subgroup analysis was limited by a smaller number of patients and thus higher standard errors.

The high rate of treatment limitations in the validation cohort deserves special attention. A DNR/DNI order was placed in a third of these patients with stroke-associated infection at a median of two days after admission. Such treatment limitations may influence clinical outcome [27, 28]. In the validation cohort, patients with treatment limitations were significantly older, had a higher burden of premorbid disability, more severe strokes and more severe stroke-associated infections, and a worse functional outcome. As these patients may have introduced some bias, the test validity of the S-SOFA score was also analyzed after excluding patients with treatment limitations. The results were similar as in the whole cohort, indicating that the high rate of treatment limitations was not a critical confounder.

A limitation of this study is that all data except the outcome at 3 months was evaluated retrospectively. Addressing the causal relationship between sepsis and poor outcome is difficult in a retrospective setting. This would require a prospective study with adjudication if poor outcome occurred due to sepsis or due to other factors such as the underlying stroke.

## Conclusions

In summary, we derived and validated the S-SOFA score as modification of the SOFA score to facilitate a diagnosis of sepsis in stroke patients. We showed that the S-SOFA score might predict poor outcome after stroke-associated infection and thus possible sepsis equally well as the SOFA score in patients not treated on an ICU. It may thus be used to identify patients with possible sepsis who have a high risk of poor outcome and need immediate treatment for stroke-associated infection. It includes parameters that can easily be obtained in patients treated on a stroke unit and is straightforward to use. In ICU patients, the S-SOFA score was not non-inferior to the SOFA score, so the SOFA score should remain the standard in these patients.

## Electronic supplementary material

Below is the link to the electronic supplementary material. I uploaded a new version of the Supplemental Material. A sentence describing the statistical methods used was added to the footnote of Supplemental Table 3. 

Supplementary Material 1

## Data Availability

The datasets used and analysed during the current study are available from the corresponding author on reasonable request.

## Abbreviations

ASPECTS: Alberta Stroke Programme Early Computed Tomography Score
AUC: Area under the receiver operating characteristic curve
CDC: Centers for Disease Control and Prevention
CI: Confidence interval
CNS: Central nervous system
COVID-19: Coronavirus disease 2019
DNI: Do not intubate
DNR: Do not resuscitate
FIO2: fraction of inspired oxygen
ICU: Intensive care unit
MAP: Mean arterial pressure
mRS: Modified Rankin scale
NIHSS: National Institutes of Health Stroke Scale
PaO2: Partial arterial oxygen pressure
qSOFA: Quick Sequential Organ Failure Assessment
SOFA: Sequential Organ Failure Assessment
S-SOFA: Stroke-Sequential Organ Failure Assessment
SpO2: Peripheral oxygen saturation
