# Supplementary material for: Derivation and validation of a screening tool for stroke-associated sepsis
Source: Neurol Res Pract. 2023 Jul 13;5:32. doi: 10.1186/s42466-023-00258-4 (PMC10339529; doi:10.1186/s42466-023-00258-4)
Supplement: Supplementary file 1 — Supplementary Material 1 [file 42466_2023_258_MOESM1_ESM.pdf]

SUPPLEMENTAL MATERIAL

Derivation and validation of a screening tool for stroke-associated sepsis

Sebastian Stösser[a], Lisa Kleusch[a], Alina Schenk[b], Matthias Schmid[b], Gabor C. Petzold[ac]

[a]Division of Vascular Neurology, Department of Neurology, University Hospital Bonn, Germany
[b]Institute of Medical Biometry, Informatics and Epidemiology, Medical Faculty, University of Bonn, Germany
[c]German Center for Neurodegenerative Diseases (DZNE), Bonn, Germany

Supplemental Figure 1: Flowchart illustrating the screening process for the validation cohort

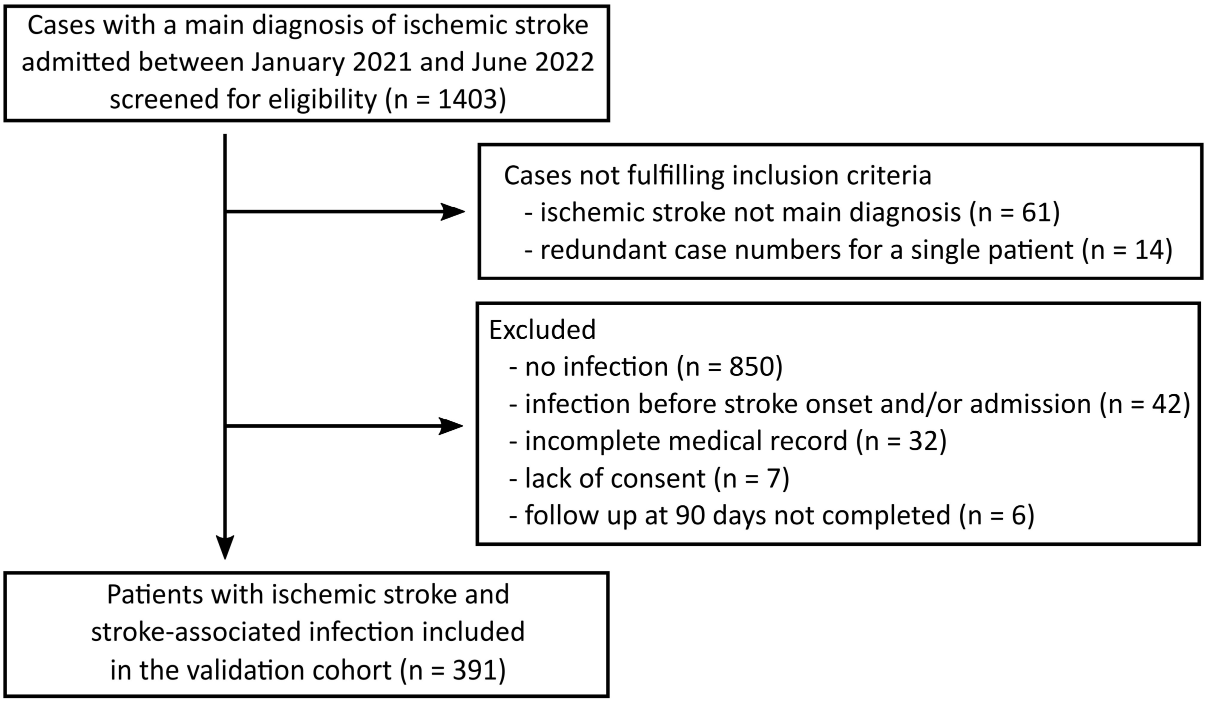

Supplemental Table 1: Cutoffs for alternative predictors of SOFA score subcategory variables and additional cut-offs chosen for further analysis based on clinical considerations

Predictor	Cutoff derived using the Youden-Index	Additional cutoff chosen for further analysis
NIHSS at 24 hours	≥14	
NIHSS subcategory “level of consciousness”	≥1	
GCS	<13	
Respiratory rate, per min	≥32	
Peripheral oxygen saturation (S_pO_2), %	<90	
Mean arterial pressure, mmHg	<65	<70
Thrombocyte count, 10^9/l	<175	<150
Creatinine, mg/dl	≥1.1	≥1.2

Abbreviations: SOFA = Sequential Organ Failure Assessement, NIHSS = National Institutes of Health Stroke Scale, GCS = Glasgow Coma Scale

Supplemental Table 2: Different combinations of the derived predictors and their goodness of fit indicated by Nagelkerke's R^2

Model	CNS	Respiration	Cardiovascular	Coagulation	Renal	R^2
1	NIHSS at 24 h	Respiratory rate	MAP < 65	Thrombocytes	Creatinine	0.315
2	NIHSS at 24 h	Respiratory rate	MAP < 65	Thrombocytes	-	0.302
3	NIHSS at 24 h	Respiratory rate	MAP < 70	Thrombocytes	Creatinine	0.312
4	NIHSS at 24 h	Respiratory rate	MAP < 70	Thrombocytes	-	0.300
5	NIHSS at 24 h	Respiratory rate	-	Thrombocytes	Creatinine	0.294
6	NIHSS at 24 h	Respiratory rate	-	Thrombocytes	-	0.281
7	NIHSS at 24 h	S_pO_2	MAP < 65	Thrombocytes	Creatinine	0.310
8	NIHSS at 24 h	S_pO_2	MAP < 65	Thrombocytes	-	0.294
9	NIHSS at 24 h	S_pO_2	MAP < 70	Thrombocytes	Creatinine	0.310
10	NIHSS at 24 h	S_pO_2	MAP < 70	Thrombocytes	-	0.295
11	NIHSS at 24 h	S_pO_2	-	Thrombocytes	Creatinine	0.300
12	NIHSS at 24 h	S_pO_2	-	Thrombocytes	-	0.283
13	NIHSS consciousness	Respiratory rate	MAP < 65	Thrombocytes	Creatinine	0.188
14	NIHSS consciousness	Respiratory rate	MAP < 65	Thrombocytes	-	0.174
15	NIHSS consciousness	Respiratory rate	MAP < 70	Thrombocytes	Creatinine	0.186
16	NIHSS consciousness	Respiratory rate	MAP < 70	Thrombocytes	-	0.172
17	NIHSS consciousness	Respiratory rate	-	Thrombocytes	Creatinine	0.181
18	NIHSS consciousness	Respiratory rate	-	Thrombocytes	-	0.166
19	NIHSS consciousness	S_pO_2	MAP < 65	Thrombocytes	Creatinine	0.196
20	NIHSS consciousness	S_pO_2	MAP < 65	Thrombocytes	-	0.180
21	NIHSS consciousness	S_pO_2	MAP < 70	Thrombocytes	Creatinine	0.196
22	NIHSS consciousness	S_pO_2	MAP < 70	Thrombocytes	-	0.180
23	NIHSS consciousness	S_pO_2	-	Thrombocytes	Creatinine	0.195
24	NIHSS consciousness	S_pO_2	-	Thrombocytes	-	0.179
25	GCS	Respiratory rate	MAP < 65	Thrombocytes	Creatinine	0.222
26	GCS	Respiratory rate	MAP < 65	Thrombocytes	-	0.203
27	GCS	Respiratory rate	MAP < 70	Thrombocytes	Creatinine	0.221
28	GCS	Respiratory rate	MAP < 70	Thrombocytes	-	0.202
29	GCS	Respiratory rate	-	Thrombocytes	Creatinine	0.221
30	GCS	Respiratory rate	-	Thrombocytes	-	0.201
31	GCS	S_pO_2	MAP < 65	Thrombocytes	Creatinine	0.227
32	GCS	S_pO_2	MAP < 65	Thrombocytes	-	0.206
33	GCS	S_pO_2	MAP < 70	Thrombocytes	Creatinine	0.227
34	GCS	S_pO_2	MAP < 70	Thrombocytes	-	0.206
35	GCS	S_pO_2	-	Thrombocytes	Creatinine	0.227
36	GCS	S_pO_2	-	Thrombocytes	-	0.206

Abbreviations: NIHSS = National Institutes of Health Stroke Scale, GCS = Glasgow Coma Scale, MAP = mean arterial pressure in mmHg

Supplemental Table 3: Comparison of patients in the validation cohort with and without treatment limitations

**Baseline data**	No treatment limitations (n = 228)	Treatment limitations (n = 163)	p-value
Age, year, median (Q1-Q3)	76 (68.3-83)	84 (75-89)	**<0.001**
Sex, female	122 (53.5)	98 (60.1)	0.194
Arterial hypertension	193 (84.6)	142 (87.1)	0.492
Dyslipidemia	160 (70.2)	105 (64.4)	0.230
Atrial fibrillation	99 (43.4)	101 (62.0)	**<0.001**
Smoking	54 (23.7)	24 (14.7)	**0.029**
Diabetes mellitus	80 (35.1)	45 (27.6)	0.118
Charlson comorbidity index score, median (Q1-Q3)	1 (1-2.8)	2 (1-3)	0.521
Premorbid mRS, median (Q1-Q3)	0 (0-3)	2 (0-3)	**<0.001**
**Stroke characteristics and treatment**			
NIHSS score at admission, median (Q1-Q3)	7 (4-13)	16 (9-20)	**<0.001**
ASPECTS at admission, median (Q1-Q3)	7 (5.8-9)	6 (4.5-8)	0.096
Occlusion of large intracranial arteries	100 (43.9)	103 (63.2)	**<0.001**
Middle cerebral artery, M1 segment	41 (41.0)	47 (45.6)	0.055[a]
Middle cerebral artery, M2 segment	25 (25.0)	27 (26.2)	0.432[a]
Intracranial internal carotid artery	17 (17.0)	18 (17.5)	0.663[a]
Basilar artery	7 (7.0)	6 (5.8)	0.740[a]
Other	10 (10.0)	5 (4.9)	>0.999[a]
Stroke etiology			**0.002**
Cardioembolism	72 (31.6)	83 (50.9)	**<0.005[a]**
Large artery arteriosclerosis	43 (18.9)	18 (11.0)	0.108[a]
Small vessel disease	13 (5.7)	6 (3.7)	0.72[a]
Other determined etiology	9 (3.9)	8 (4.9)	0.646[a]
Undetermined etiology	91 (39.9)	48 (29.4)	0.132[a]
Intravenous thrombolysis	65 (28.5)	32 (19.6)	**0.045**
Endovascular therapy	70 (30.7)	75 (46.0)	**0.002**
General anesthesia for endovascular therapy	66 (94.3)	71 (95.9)	0.643
Symptomatic intracranial hemorrhage after intravenous thrombolysis or endovascular therapy	8 (7.7)	10 (11.8)	0.387
**Stroke-associated infection**			
Time from admission to diagnosis of infection, days, median (Q1-Q3)	3 (2-7)	2 (2-4)	**<0.001**
Source of infection			
Pneumonia (clinical diagnosis)	84 (36.8)	102 (62.6)	**<0.001**
Pneumonia (according to PISCES criteria)	59 (25.9)	76 (46.6)	**<0.001**
Urinary tract infection (clinical diagnosis)	111 (48.7)	59 (36.2)	**0.014**
Urinary tract infection (according to CDC criteria)	11 (4.8)	7 (4.3)	0.805
Other	27 (11.8)	4 (2.5)	**<0.001**
Undetermined	22 (9.6)	19 (11.7)	0.523
COVID-19	11 (4.8)	6 (3.7)	0.585
Evidence of a pathogenic organism in body fluid cultures	118 (51.8)	80 (49.1)	0.602
Antibiotic therapy	217 (95.2)	153 (93.9)	0.571

Time from diagnosis of infection to antibiotic therapy, hours, median (Q1-Q3)	1.5 (0.2-5.4)	1.3 (0-3.8)	0.116
Sepsis (diagnosis according to Sepsis-3 definition)	53 (23.2)	76 (46.6)	**<0.001**
**Clinical outcome at discharge**			
NIHSS, median (Q1-Q3)	4 (2-7.5)	11.5 (5-17.3)	**<0.001**
mRS, median (Q1-Q3)	4 (2-5)	5 (5-6)	**<0.001**
Barthel index, median (Q1-Q3)	45 (20-75)	10 (0-20)	**<0.001**
Length of stay, d, median (Q1-Q3)	14 (8-20)	10 (5-18)	**<0.001**
ICU treatment	50 (21.9)	45 (27.6)	0.197
Death	5 (2.2)	67 (41.1)	**<0.001**
**Clinical outcome at 3 months**			
mRS, median (Q1-Q3)	4 (3-5)	6 (5-6)	**< 0.001**
mRS 0	4 (1.8)	0 (0.0)	0.088[a]
mRS 1	21 (9.3)	0 (0.0)	**<0.010[a]**
mRS 2	19 (8.4)	1 (0.6)	**<0.010[a]**
mRS 3	51 (22.5)	5 (3.1)	**<0.010[a]**
mRS 4	63 (27.8)	24 (14.7)	**<0.010[a]**
mRS 5	44 (19.3)	16 (9.8)	**0.020[a]**
Death (mRS 6)	25 (11.0)	117 (71.8)	**<0.010[a]**
Functional independency (mRS 0-2)	44 (19.4)	1 (0.6)	**<0.010[a]**
Unfavourable outcome (mRS 4-6)	132 (58.1)	157 (96.3)	**<0.010[a]**
Poor outcome (mRS 5-6)	69 (30.3)	133 (81.6)	**<0.010[a]**

Results are presented as n (%) unless indicated otherwise for certain variables. Differences in metric data were assessed using the Mann-Whitney U test; differences in frequencies were assessed using the Pearson chi-square test. Bold type indicates p values smaller than the significance level α = 0.05. ‘[a]’ indicates p values that were adjusted for multiple comparisons using the Bonferroni-Holm method.
Abbreviations: ASPECTS = Alberta Stroke Programme Early Computed Tomography Score, CDC = Centers for Disease Control and Prevention, COVID-19 = coronavirus disease 2019, DNI = Do not intubate, DNR = Do not resuscitate, ICU = intensive care unit. mRS = modified Rankin Scale, NIHSS = National Institutes of Health Stroke Scale, PISCES = Pneumonia in Stroke Consensus. Other occlusions of large intracranial arteries include vertebral artery, P1/2 segment of the posterior cerebral artery, and A1 segment of the anterior cerebral artery.

Supplemental Table 4: Area under the receiver operating curve (AUC) of S-SOFA and SOFA scores for the prediction of an unfavourable outcome, defined by a value of 4 to 6 on the modified Rankin scale, and their difference with 95% confidence intervals

Cohort	S-SOFA score	SOFA score	Difference	p-value for non-inferiority
Full (n = 390)	0.671 [0.615; 0.727]	0.752 [0.702; 0.801]	-0.081 [-0.12; -0.042]	0.854
No treatment limitations (n = 227)	0.613 [0.543; 0.683]	0.686 [0.62; 0.752]	-0.073 [-0.122; -0.024]	0.697
ICU treatment (n = 95)	0.756 [0.631; 0.882]	0.843 [0.723; 0.964]	-0.087 [-0.193; 0.019]	0.693
ICU treatment and no treatment limitations (n = 50)	0.751 [0.602; 0.899]	0.82 [0.681; 0.96]	-0.07 [-0.194; 0.054]	0.561
Non-ICU treatment (n= 295)	0.636 [0.572; 0.701]	0.706 [0.647; 0.765]	-0.069 [-0.112; -0.027]	0.669
Non-ICU treatment and no treatment limitations (n = 178)	0.559 [0.479; 0.64]	0.618 [0.539; 0.697]	-0.059 [-0.114; -0.004]	0.485

If the lower limit of the 95% confidence interval of the difference was greater than or equal to -0.060 (or, equivalently, if the p-value was less than 0.025), the null-hypothesis of inferiority was rejected, and non-inferiority of the S-SOFA score was assumed.
Abbreviations: ICU = intensive care unit, SOFA = Sequential Organ Failure Assessment, S-SOFA = Stroke-SOFA

Supplemental Material 1: Sample Size Calculation

For sample size calculation, an effect size of $\delta = 0.06$ and a non-inferiority margin of $\Delta = 0.06$ was assumed. Let AUC_1 be the AUC of the SOFA score and AUC_2 be the AUC of the Stroke SOFA score (S_SOFA in the formulae). According to the method of DeLong [10, 17] the standard error of the difference of the AUC values is given through

$$SE(AUC_2 - AUC_1) = \sqrt{Var(AUC_2) + Var(AUC_1) - 2 \cdot Cov(AUC_1, AUC_2)}$$

with

$$Var(AUC_1) = \frac{Var(V_{A1})}{n_A} + \frac{Var(V_{N1})}{n_N}$$

$$Var(AUC_2) = \frac{Var(V_{A2})}{n_A} + \frac{Var(V_{N2})}{n_N}$$

$$Cov(AUC_1, AUC_2) = \frac{Cov(V_{A1}, V_{A2})}{n_A} + \frac{Cov(V_{N1}, V_{N2})}{n_N}$$

and

$$V_{A1}^{i} = \frac{1}{n_N} \sum_{j=1}^{n_N} Ranking[SOFA(i), SOFA(j)]$$

$$V_{N1}^{j} = \frac{1}{n_A} \sum_{i=1}^{n_A} Ranking[SOFA(i), SOFA(j)]$$

$$V_{A2}^{i} = \frac{1}{n_N} \sum_{j=1}^{n_N} Ranking[S\_SOFA(i), S\_SOFA(j)]$$

$$V_{N1}^{j} = \frac{1}{n_A} \sum_{i=1}^{n_A} Ranking[S\_SOFA(i), S\_SOFA(j)]$$

with

$$Ranking[SOFA(i), SOFA(j)] = \begin{cases} 0, & SOFA(i) < SOFA(j) \\ 0.5, & SOFA(i) = SOFA(j) \\ 1, & SOFA(i) > SOFA(j) \end{cases}$$

and

$$Ranking[S\_SOFA(i), S\_SOFA(j)] = \begin{cases} 0, & S\_SOFA(i) < S\_SOFA(j) \\ 0.5, & S\_SOFA(i) = S\_SOFA(j) \\ 1, & S\_SOFA(i) > S\_SOFA(j) \end{cases}$$

where $i = 1, \ldots, n_A$ refers to the individuals with poor outcome (outcome = 1, n_A in total) and $j = 1, \ldots, n_N$ refers to the individuals without poor outcome (outcome = 0, n_N in total). For a

given ratio $\frac{n_N}{n_A} = m$ of individuals without and with poor outcome, and using the relation $Cov(X,Y) = Corr(X,Y) \cdot \sqrt{Var(X)Var(Y)}$, sample size was obtained via

$$n_A > \frac{\sigma^2 \cdot \left(z_{1-\frac{\alpha}{2}} - z_\beta\right)^2}{(\delta + \Delta)^2 \cdot m},$$

where

$$\sigma^2 = m \cdot Var(V_{A1}) + Var(V_{N1}) + m \cdot Var(V_{A2}) + Var(V_{N2}) + m \cdot Corr(V_{A1}, V_{A2})\sqrt{Var(V_{A1})Var(V_{A2})} + Corr(V_{N1}, V_{N2})\sqrt{Var(V_{N1})Var(V_{N2})}.$$

For our purposes, we assumed $\alpha = 0.05, \beta = 0.2$ (probability of β error, equivalent to a power of 80%), $\delta = \Delta = 0.06, m = 2.\overline{3}$ (which is equivalent to 30% of patients with poor outcome), $Var(V_{A1}) = Var(V_{N1}) = Var(V_{A2}) = Var(V_{N2}) = 0.05, Corr(V_{A1}, V_{A2}) = Corr(V_{N1}, V_{N2}) = 0.7$.

Supplemental Material 2: Details on the non-inferiority test

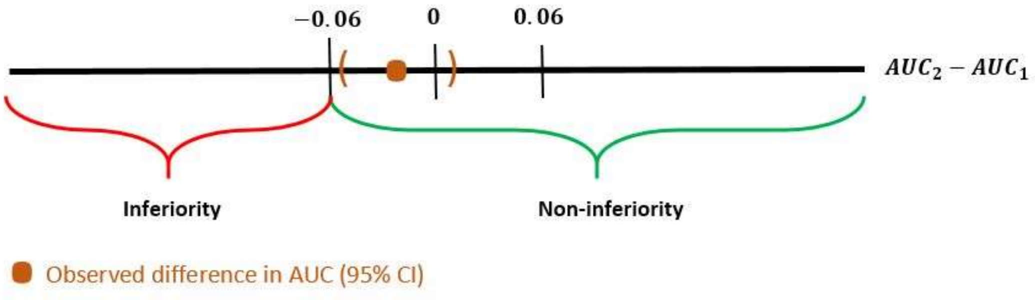

95% confidence intervals for the difference of AUC are constructed as $AUC_{S-SOFA} - AUC_{SOFA} \mp z_{\frac{\alpha}{2}} \cdot SE(AUC_{S-SOF} \quad - AUC_{SOFA})$, where AUC_{SOFA} (AUC_{S-SOFA}) refers to the AUC of the (modified) SOFA score on the validation cohort. See Supplemental Material 1 for the calculation of $SE(AUC_{S-SOF} \quad - AUC_{SOFA})$. The null-hypothesis of inferiority of the modified SOFA score was rejected if the lower bound of the confidence interval for the difference is larger than -0.06 or, equivalently, if the respective p-value, given as $1 - \Phi\left(\frac{AUC_{S-SOF} \quad - AUC_{SOFA} + 0.06}{SE(AUC_{S-SOF} \quad - AU \quad _{SOFA})}\right)$, with Φ being the cumulative distribution function of the standard normal distribution, is smaller than 0.025.
